# Cortical Visual Evoked Potentials and Growth in Infants Fed with Bioactive Compounds-Enriched Infant Formula: Results from COGNIS Randomized Clinical Trial

**DOI:** 10.3390/nu11102456

**Published:** 2019-10-14

**Authors:** Ana Nieto-Ruiz, José Antonio García-Santos, Mercedes G. Bermúdez, Florian Herrmann, Estefanía Diéguez, Natalia Sepúlveda-Valbuena, Salomé García, Maria Teresa Miranda, Roser De-Castellar, María Rodríguez-Palmero, Andrés Catena, Cristina Campoy

**Affiliations:** 1 EURISTIKOS Excellence Centre for Paediatric Research, Biomedical Research Centre, University of Granada, 18016 Granada, Spain; ananietoruiz@gmail.com (A.N.-R.); joseantonio_gsantos@outlook.es (J.A.G.-S.); mgbermudez@ugr.es (M.G.B.); dr.f.herrmann@gmail.com (F.H.); estefaniadieguezcastillo@gmail.com (E.D.); sepulveda.natalia@hotmail.com (N.S.-V.); 2 Department of Paediatrics, School of Medicine, University of Granada, Avda. Investigación 11, 18016 Granada, Spain; 3 Mind, Brain and Behaviour Research Centre-CIMCYC, University of Granada, 18011 Granada, Spain; acatena@ugr.es; 4 Nutrition and Biochemistry Department, Faculty of Sciences, Pontificia Universidad Javeriana, Bogotá 110231, Colombia; 5 Clinical Service of Neurophysiology, Clinical University Hospital San Cecilio, 18016 Granada, Spain; salomegarcia2@yahoo.es; 6 Department of Biostatistics, School of Medicine, University of Granada, 18016 Granada, Spain; tmiranda@ugr.es; 7 Ordesa Laboratories, S.L., 08820 Barcelona, Spain; Roser.DeCastellar@ordesa.es (R.D.-C.); Maria.Rodriguez@ordesa.es (M.R.-P.); 8 Spanish Network of Biomedical Research in Epidemiology and Public Health (CIBERESP), Granada’s node, Institute of Health Carlos III, 28029 Madrid, Spain

**Keywords:** breastfeeding, infant formula, bioactive compounds, visual evoked potentials, growth

## Abstract

Postnatal nutrition is essential for growth and neurodevelopment. We analyzed the influence of a new enriched-infant formula with bioactive compounds on growth, neurodevelopment, and visual function (VF) in healthy infants during their first 18 months of life. A total of 170 infants were randomized in the COGNIS randomized clinical trial (RCT) to receive a standard infant formula (SF = 85) or a new experimental infant formula supplemented with functional nutrients (EF = 85). As a control, 50 breastfed infants (BF) were enrolled. Growth patterns were evaluated up to 18 months of life; neurodevelopment was assessed by general movements at 2, 3, and 4 months; VF was measured by cortical visual evoked potentials at 3 and 12 months. No differences in growth and neurodevelopment were found between groups. Regarding VF, SF and EF infants presented prolonged latencies and lower amplitudes in the P100 wave than BF infants. In the EF group, a higher percentage of infants presented response at 7½′ of arc at 12 months compared to 3 months of age; a similar proportion of BF and EF infants presented responses at 7½′ of arc at 12 months of age. Early nutritional intervention with bioactive compounds could narrow the gap in growth and neurodevelopment between breastfed and formula-fed infants.

## 1. Introduction

Early postnatal nutrition is one of the major determinants of healthy growth and development during infancy. Human milk has a variety of health benefits on growth, brain development, and immune system function, among others [1]. When breastfeeding is not possible, infant formulas are the substitute of choice. These infant formulas try to mimic the nutritional composition of breast milk as closely as possible [2].

Deficiency or excess of certain nutrients would determine an inadequate growth pattern during early life [3]. It is known that protein intake during early life is associated with more rapid weight gain during infancy. In the Childhood Obesity Program (CHOP) study, authors found a higher risk of developing obesity in the higher protein-content infant formula group when evaluated the effect of different protein-content infant formula on body mass index (BMI) at 6 years of age [4].

Differences in long-chain polyunsaturated fatty acids (LC-PUFAs) content in breast milk and infant formula have also been hypothesized to affect infant growth and development [5]. LC-PUFAs, in particular, docosahexaenoic acid (DHA) and arachidonic acid (AA), are necessary for synaptogenesis, myelination, and growth and maturation of infants’ brain and retina [6,7]. Deficiencies of LC-PUFAs have been associated with impairments in cognitive performance. The supplementation with LC-PUFAs during pregnancy and lactation has been proposed to prevent nutritional deficiencies in the offspring. Taking this into account, the majority of commercialized infant formulas are enriched with LC-PUFAs [8]. Nonetheless, according to the available evidence, the results are inconclusive and need to be analyzed in the long-term [6,8,9].

Recently, the supplementation of infant formulas with bovine milk fat globule membrane (MFGM) has emerged as a possible way to improve infant health. This membrane fraction is composed of different bioactive components (phosphatidylcholine, sphingomyelin, and cholesterol, as well as cerebrosides, gangliosides, glycosylated proteins and polypeptides, filaments, mucins, and so on) [10]. It has been demonstrated that MFGM has positive effects on both neurodevelopment and defense against infections, especially in formula-fed infants, since infant formulas traditionally have lower concentrations of biologically active MFGM in comparison to breast milk [11]. Timby et al. observed that infants fed with MFGM supplemented formulas had similar cognitive development and growth patterns at 12 months of age compared to those who were breastfed [12].

Synbiotics, a combination of pre- and probiotics, are being also added to new infant formulas as some positive effects on intestinal immune response have been observed [13]. It is known that the intestinal microbiota might contribute to the shaping of neural networks and the response to neurotransmitters [14].

Having in mind these considerations, the current study analyzed the effects of new infant formula, supplemented with functional nutrients, on growth, neurodevelopment, and visual function in healthy infants during their first 18 months of life, compared to those fed a standard formula or breast-fed.

## 2. Materials and Methods

### 2.1. Ethics, Consent, and Permissions

This study was carried out following the updated Declaration of Helsinki Principles [15], the Good Clinical Practice recommendations of the EEC (document 111/3976/88 July 1990), as well as the current Spanish legislation governing clinical research in humans (Royal Decree 561/1993 on clinical trials). The study protocols were also approved by the Research Bioethical Committee from the University of Granada, and the Bioethical Committees for Clinical Research of the Clinical University Hospital San Cecilio and the Mother-Infant University Hospital of Granada (Granada, Spain). All families were informed about procedures, and a signed written informed consent was obtained from each parent or legal guardian for their offspring.

### 2.2. Study Design and Subjects

**Figure 1:**
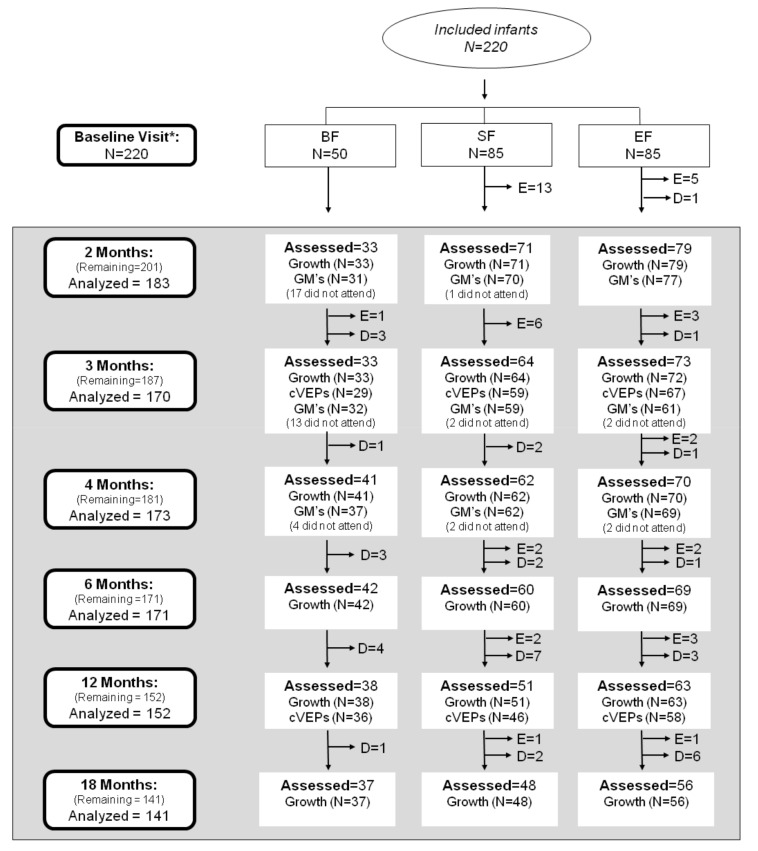
Study profile from baseline visit to 18 months old. Eighteen infants at 2 months, seventeen at 3 months, and eight at 4 months did not show up at the evaluations but remained in COGNIS study for later visits (described as “did not attend”). BF: breastfed infants; SF: standard infant formula; EF: experimental infant formula; D: dropouts; E: exclusions; GM’s: general movements; cVEPs: cortical visual evoked potentials. *BF infants were randomized between 0–6 months of age.

The COGNIS study (A Neurocognitive and Immunological Study of a New Formula for Healthy Infants) is designed as a prospective, randomized double-blind, nutritional intervention study registered at www.ClinicalTrials.gov, Identifier NTC02094547. This study was aimed to evaluate the effects of a new infant formula enriched with bioactive components on child neurocognitive, growth, and immunological development, compared to those infants who received a standard infant formula or were breastfed. The design of the COGNIS study has been published elsewhere [16]. Briefly, 220 full-term infants were recruited at the EURISTIKOS Excellence Center for Pediatric Research, University of Granada (Spain), through collaboration with local health centers. Of them, 170 infants aged 0–2 months old were randomized to fed, during their first 18 months of life, standard infant formula (SF; *n* = 85) or experimental infant formula (EF; *n* = 85) supplemented with bioactive compounds, including MFGM components (10% of total protein content (wt:wt)), synbiotics (mix of fructooligosaccharides (FOS) and inulin (ratio 1:1); *Bifidobacterium infantis* IM1 and *Lactobacillus rhamnosus* LCS-742), LC-PUFAs (AA and DHA), gangliosides, nucleotides and sialic acid. A full description of the nutritional composition of both infant formulas has been previously reported [17]. Moreover, 50 infants who were exclusively breastfed (BF) for at least 2 months were recruited from 0–6 months of age and included as a control group. Detailed study profile and follow-up of the COGNIS participants up to 18 months of life are shown in Figure 1.

### 2.3. Data Collection

Baseline information about maternal and paternal age, pre-gestational body mass index (BMI), a gain of weight during pregnancy, siblings, type of delivery, smoking habits during pregnancy, as well as parents’ educational levels, place of residence and intelligence quotient (IQ) was obtained at study entry. Information regarding the newborn, including gestational age, sex, and anthropometric characteristics, was also registered.

Children were followed-up at 2, 3, 4, 6, 12, and 18 months of life for anthropometric evaluation, while general movements’ (GM’s) assessment was performed at 2, 3, and 4 months life; cortical visual evoked potentials (cVEP) measures were performed at 3 and 12 months of life.

### 2.4. Assessments of Infant and Children Growth

Infant anthropometric characteristics (weight, length, and subsequent body mass index (BMI)) were analyzed in order to evaluate the effect of EF on growth patterns. Infant weight was obtained by using the baby scale "Multina Comfort" 8352 Soehnle-Professional (max 20 kg). Holtain Harpenden Infantometer Model 702 (max 91.5 cm) was used to measure infant length. All anthropometric parameters were assessed according to the World Health Organization (WHO) growth standards 2006–2007 by sex and age [18], and z-scores for weight/age (WAZ), weight/length (WLZ), length/age (LAZ), and BMI/age (BAZ) were calculated using the available software for the WHO growth standards (WHO Anthro software, version 3.2.2, World Health Organization, Geneva, Switzerland).

### 2.5. Neurological Development: General Movements (GM’s) Test

General movements (GM’s) test was performed at 2, 3, and 4 months of age. GM’s test at the early stages of life is predictive of motor and cognitive development in term infants. This test measures a series of gross movements of variable amplitude and speed involving all body parts; evaluation of such movements includes the quality of GM’s (amount of movement variation, complexity, and fluency) exhibited by the infant, using a video recording of the baby [19].

This procedure can distinguish four kinds of GM’s based on complexity, variation, and fluidity of movement: two normal (normal-optimal, normal-suboptimal) and two abnormal (moderately abnormal and definitively abnormal) (Table 1) [19].

### 2.6. Visual Function: Cortical Visual Evoked Potentials (cVEPs)

Infants’ cVEPs were recorded at 3 and 12 months of age to obtain an objective measure about their visual function, which is considered an optimal indicator of general neurodevelopment in the infant population [20]. Measurement of cVEPs in COGNIS infants was performed, as previously described [21]. Briefly, infants’ evaluation was conducted in a partially dark, quiet room under controlled conditions. Long-lasting, reusable elastic spandex-type caps, with age-adjusted sizes, and electrodes placed according to standard international 10–20 positioning system, were used. Schwarzertopas EMG System (NATUS, California, USA) was used to register infants’ cVEPs. Visual stimuli (reversal pattern of a black and white checkerboard with 100% contrast) were performed in a shape of binocular frequencies at 2°, 1°, 30′, 15′, and 7½′. The average luminance was 39 kcd/m^2^, and the investment rate was 2.1. Responses were amplified with a filter from 1.5 Hz to 100 Hz. cVEPs measurement was replicated in those cases where the infant did not pay attention to the stimulus. In the current study, P100 wave latencies and amplitudes were used, and visual function was evaluated by the response at a minimum angle of resolution [21].

### 2.7. Statistical Analysis

The sample size required was calculated before the beginning of the study using a standard approach; to detect a minimum difference of 0.5 standard deviations (SD) in cVEPs with a statistical power of 90% and α = 0.05, the sample size was established in 71 infants/formula group. However, having in mind a potential dropout rate of 20%, the final sample size was estimated in 85 infants/formula group.

All statistical analyses were performed using the SPSS statistical software package (version 22.0; IBM SPSS Inc., Chicago, IL, USA). Continuous, normally distributed variables were expressed as mean and standard deviation (SD); non-continuous variables were presented as the median and interquartile range (IQR); thus, appropriate nonparametric tests were used for their statistical analysis. Unadjusted analysis of variance (ANOVA), adjusted multivariate analysis of covariance (ANCOVA), as well as generalized linear mixed model (GLMM) for repeated measures, were developed to identify differences between study groups. For categorical variables, appropriate tests, depending on the response variable, were used (χ^2^ or Fisher exact test). Finally, the McNemar test was performed to compare proportions in infant visual function. *p*-values <0.05 were considered as statistically significant.

## 3. Results

### 3.1. Parental and Newborn Characteristics of the COGNIS Study Participants

Of the 220 infants included in the COGNIS study, one dropped out of the study, and 18 were excluded before the first follow-up visit at 2 months of age, primarily due to not meeting inclusion criteria. From the initial visit to 18 months, one infant was excluded in the BF group due to not receiving exclusive breastfeeding. A total of 40 infants were excluded in the SF and EF groups: 24 were excluded in the SF group (one infant due to perinatal hypoxia, one infant had growth deficiency, 15 infants who did not ingest infant formula, two had colic, three infants were lactose intolerant, one infant had digestive surgical intervention, and one infant suffered hydrocephalus); 16 infants were excluded in the EF group (two infants presented growth deficiency, two infants were lactose intolerant, 11 infants did not ingest infant formula, and one infant had epileptic seizure). Infants dropouts were mainly due to not attending the visits or change of residence (Figure 1).

The baseline characteristics of participants are shown in Table 2. Significant differences in maternal IQ and parent’s educational level were found between COGNIS study groups. Maternal IQ from the BF group was significantly higher than those who gave infant formula to their infants (*p* < 0.001). Furthermore, parents of BF infants presented higher educational levels in comparison with parents of SF and EF fed infants (maternal *p* < 0.001; paternal *p* = 0.003). No significant differences were found in other baseline characteristics (parent’s age, maternal pre-conceptional BMI, gestational age, weight gain during pregnancy, and siblings) between the three study groups.

Regarding newborn characteristics, no differences between study groups were found in the type of delivery, or infant anthropometric data, including weight, length, and head circumference at birth. Due to the COGNIS study design, days of breastfed significantly differed between study groups (*p* < 0.001).

### 3.2. Effects of Experimental Formula on Infant’s Growth Up to 18 Months of Life

**Figure 2:**
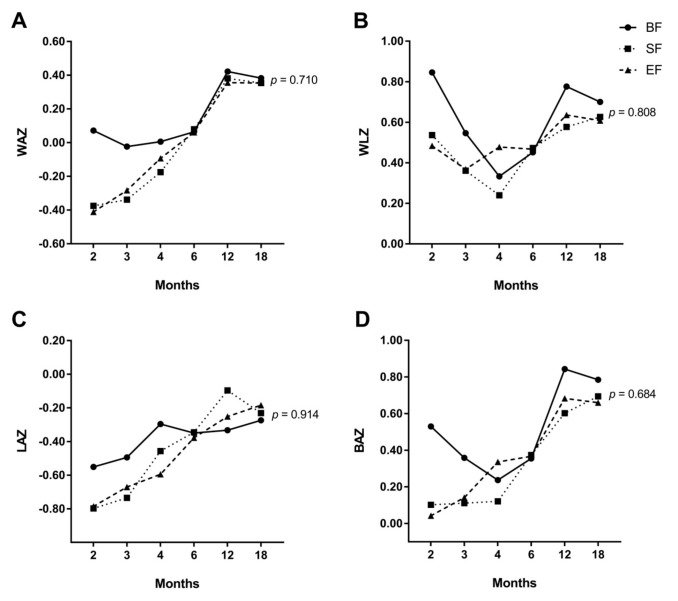
Generalized linear mixed model of repeated measures for z-scores for weight/age (WAZ) (**A**), weight/length (WLZ) (**B**), length/age (LAZ) (**C**), and body mass index (BMI)/age (BAZ) (**D**) in COGNIS infants. BF: breastfeeding; SF: standard infant formula; EF: experimental infant formula.

In order to evaluate the effects of experimental formula on infants’ growth compared to standard formula or breast milk, a longitudinal study of growth up to 18 months of age was conducted using a general linear mixed model of repeated measures (Supplementary Table S1). No differences between study groups were found in WAZ (*p* = 0.710) (Figure 2A), WLZ (*p* = 0.808) (Figure 2B), LAZ (*p* = 0.914) (Figure 2C), and BAZ (*p* = 0.684) (Figure 2D) during the first 18 months of age. However, our results seemed to suggest a similar healthy growth pattern in both infant formula groups compared to BF in terms of WAZ from 6 to 18 months of age. Furthermore, both EF and BF infants at 6–18 months of age seemed to follow a similar growth profile in WLZ and BAZ. Z-scores achieved were higher in BF infants at 0 – 2 months of life than those obtained in formula-fed infants. However, after 6 months of age, all of the z-scores converged and did not show statistically significant differences between groups.

### 3.3. Results from GM’s

Neurological development was evaluated at 2, 3, and 4 months of life using the quality of GM’s. All infants presented an adequate neurological development up to 4 months of life, and no statistically significant differences were found between the COGNIS group at any point in time (Table 3).

### 3.4. Results from Cortical Visual Evoked Potentials Examination

We next evaluated the influence of EF on visual function during the first 12 months of life, compared to SF and breastfeeding. For this purpose, cVEPs were assessed at 3 and 12 months of life (Table 4). At 3 months of life, BF infants presented shorter latencies at 60′ (*p* = 0.012), 30′ (*p* = 0.015), and 15′ (*p* = 0.040) of arc compared to EF infants, after adjusting for confounders (maternal educational level and IQ, and paternal educational level). Conversely, we found higher amplitudes in infants who were breastfed compared to those who received EF or SF in the P100 wave at 60′ (adjusted *p* = 0.007), 30′ (adjusted *p* = 0.014), and 15′ of arc (adjusted *p* = 0.005). The results of the cVEPs performed at 3 months did not show significant differences between EF and SF infants.

At 12 months of age, adjusted analysis showed that BF infants had shorter latencies at 15′ of arc (adjusted *p* = 0.004) compared to those who were fed with EF or SF, while there were significant differences between BF infants and EF infants with regard to the latencies obtained at 7½′ of arc (adjusted *p* = 0.031). BF infants also presented higher amplitudes at 120′ of arc compared to both SF and EF infants (adjusted *p* = 0.003) (Table 4).

**Figure 3:**
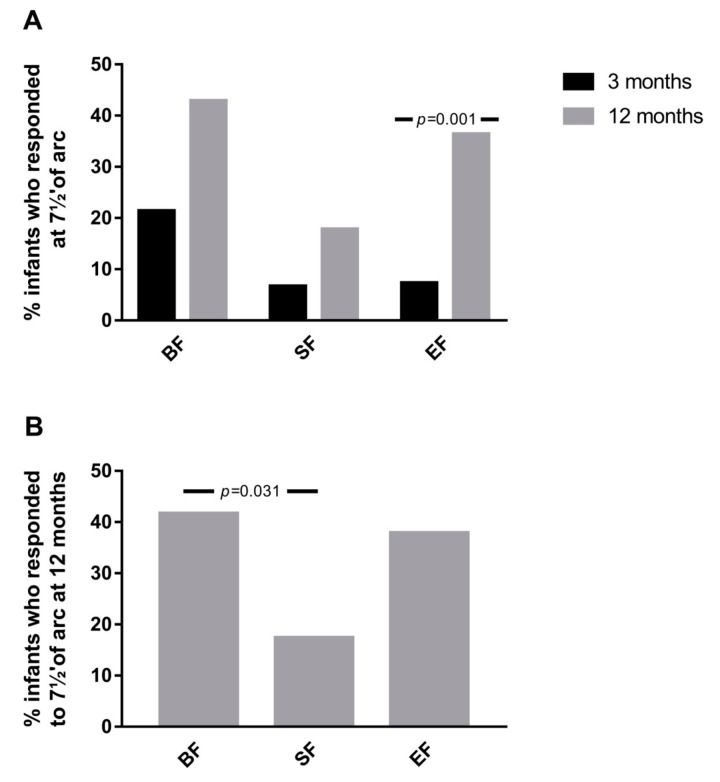
(**A**) % of infants who presented response at 7½′ of arc at 12 months of age compared to 3 months of age in the three study groups. (**B**) % of infants at 12 months who showed a response at 7½′ of arc in the three study groups. *p*-values were obtained from McNemar test (A), and Chi-square test (B). SF: standard infant formula; EF: experimental infant formula; BF: breastfed infants.

To further explore the effects of experimental formula on visual function, the proportion of infants who showed response at a minimum angle of resolution (7½′ of arc) at 3 and 12 months of age was analyzed. As shown in Figure 3A, in the EF group, a higher percentage of infants presented response at 7½′ of arc at 12 months of age compared to 3 months of age (*p* = 0.001). These effects were not observed in the SF (*p* = 0.227) and BF (*p* = 0.180) infants groups. Additionally, at 12 months of age, the proportion of infants who presented response at 7½′ of arc was similar between BF and EF groups, which was higher compared to those who received SF (*p* = 0.031) (Figure 3B).

## 4. Discussion

To our knowledge, this is the first study with a nutritional intervention during the first 18 months of life, comparing the effects of a new formula enriched with a range of bioactive compounds, including MFGM, LC-PUFAs, synbiotics, gangliosides, nucleotides, and sialic acid, on infant growth, early neurodevelopment, and visual function. Our results suggested that there were no significant differences in growth between COGNIS study groups during the first 18 months of life. Regarding neurological development, infants fed SF or EF presented similar gross movements’ quality, with no differences compared to breastfed infants during the first 4 months of life. We found that BF infants had a significantly better visual function at both 3 and 12 months of life, in terms of shorter latencies and higher amplitudes than formula-fed infants. Interestingly, a further exploratory analysis showed no differences in the proportion of infants who showed a response to minimum visual stimulus between BF and EF infants at 12 months, suggesting that the new experimental formula may promote positive effects on visual function in early postnatal life.

Growing evidence supports the concept of “early nutritional programming” by which optimal nutrition during the first 1000 days of life can determine a healthy adult life, in terms of neurodevelopment, growth, metabolism, and reduced risk of common non-communicable diseases [22]. Breastfeeding is considered a gold standard of early nutrition for optimal infant development in view of a wide range of nutrients and other bioactive compounds presented in human milk [23,24]. However, the use of infant formula, either primarily or in conjunction with breastfeeding, is currently increasing in low- and high-income countries, probably due to socioeconomic and occupational factors [25]. Currently, research efforts are focused on narrowing the nutritional and functional gap between infant formula and human milk. To date, randomized clinical trials (RCTs) have supported beneficial effects on health and neurodevelopment in infants who received infant formula enriched with one single component, such as human milk oligosaccharides, MFGM, or DHA [26]. Nonetheless, long-term beneficial effects of infant formula supplementation based on a set of functional nutrients remain unclear. Having in mind these considerations, our data have an added value due to our experimental infant formula composition, including those bioactive nutrients mentioned above, and the present aim is to evaluate its effects considering the product as a whole.

An interesting observation was the absence of significant differences in infants’ growth pattern, in terms of WAZ, WLZ, LAZ, and BAZ, between experimental or standard infant formula respect to those who were breastfed up to 18 months. This finding has a particular interest as formula-fed infants classically show accelerated growth profile compared to breastfed infants from 2 months of age to the end of the first year of life [27,28,29], which in turn is related to a higher risk of obesity and metabolic syndrome later in life [30,31,32]. The above-mentioned differences in growth patterns have been related to higher protein intake in formula-fed infants, particularly after the first 2 months of life [33]. Koleztko et al. [34] reported that 2 years-old infants who received both initiation and follow-on infant formula with lower protein content during their first year of life showed lower WLZ than those who were fed with an infant formula with higher protein levels. Interestingly, authors also showed similar WLZ in low proteins infant formula and breastfed infants. Thus, the lack of differences in growth patterns reported in the current study could be because the protein content of both infant formulas used in the COGNIS study is within the minimum recommended range (1.8 g protein/100 kcal) [35,36].

Our results seem to suggest differences, but not statistically significant, in growth pattern between first and second half-year of life in COGNIS infants, when complementary feeding is introduced. First, BF infants had exceptionally higher z-scores at 2 months of life compared to those who were formula-fed, although no differences were found in both weight and length at birth between study groups. However, 6-month-old infants showed similar z-scores, independently of the type of feeding. This is consistent with results from a previous study, where infants who were breastfed, either for the first year of life or for 4–11 months, had higher rates of growth during their first 3 months of life, while those who received infant formula showed progressive rise in growth rate from 3–6 months interval [29]. From 6–18 months of life, growth profile in terms of WLZ and BAZ appeared to be similar in EF and BF infants. There was a decreasing trend in both z-scores at 18 months of life, while, conversely, SF infants’ growth increased in this age range. Based on these findings, both breastfed and EF infants might have a slower growth rate compared to standard formula-fed infants, which in turn could reduce their risk of obesity later in life. To date, no significant differences in growth have been reported in healthy infants who received formula enriched with only a single biocompound, including DHA or DHA plus AA [37], or MFGM [12], compared to those who were fed with non-enriched formula or were breastfed. We suggest that the combination of all bioactive compounds could be responsible for the above-mentioned effect since it narrows both the energetic and nutritional gap between breast milk and infant formula. However, further follow-up studies are still needed in order to clarify some of these matters, particularly regarding the growth and the age at which the adiposity rebound occurs.

Postnatal healthy nutrition is also essential for later optimal visual function and neurodevelopment by promoting selective removal of misplaced axons and subsequent development of correct neural connections [38,39]. Thus, neuropsychological evaluation in early life should be one of the primary outcomes in nutrition research in order to determine the role of certain nutrients on brain development and function [40]. Both visual function and neurodevelopment in COGNIS infants were evaluated using GM’s quality, and the results were obtained from latencies and amplitudes of the P100 wave by cVEPs. No statistical differences between groups in GM’s test during the first 4 months of life were found. On the other hand, our results suggested that BF infants showed better visual function (higher amplitudes) and neuron myelination (shorter latencies) at 3 and 12 months compared to those who received standard or experimental infant formula. An interesting observation of the present study is that infants who received experimental formula showed better visual function, in terms of percentage of infants who presented response at 7½′ of arc at 12 months of age compared to 3 months of age. Traditionally, beneficial effects of breast milk on infant visual function and neurodevelopment have been related to a higher proportion of DHA in the brain and retina of breastfed infants [8,41,42], which might be related to the role of DHA on neurogenesis, neurite growth, and neurotransmission [37]. It must be observed that EF, and no SF, is enriched with LC-PUFAs, including DHA and AA, which are essentials in growth and maturation of infants’ brain and retina [5]. However, a systematic review supports that studies evaluating the potential effects of LC-PUFAs-enriched infant formula on visual acuity showed either no significant or minor effects [37]. Interestingly, those studies that reported beneficial effects of infant formula supplemented with LC-PUFAs were unable to demonstrate an additional improvement of visual acuity with higher quantities of LC-PUFAs supplementation [43,44]. Considering that neuronal myelination is continued for the first 2 years of life, our results suggested beneficial effects at 1 year of life of EF on the myelination process that takes place during postnatal brain development. It is, however, difficult to determine whether this positive finding can be due only to LC-PUFAs or may be a consequence of the other bioactive compounds, including MFGM, nucleotides, or synbiotics.

One of the main strengths of the present study relies on its design as a prospective RCT with a long-term nutritional intervention and monitoring; contrary to other studies, current randomized nutritional intervention with infant and follow-on formulas was prolonged from birth/2 months till 18 months of age. Thus, the present study allowed us to evaluate the potential effects of bioactive compounds-enriched infant formula on neurodevelopment and growth in infants at different time points. As previously mentioned, it is important to take into account that the experimental infant formula is based on a set of bioactive compounds rather than a unique nutrient, trying to mimic breast milk composition. To our knowledge, no studies have used this type of supplementation in infant formula. Therefore, our data may provide an efficacious approach to narrow the current development gaps observed between breastfed and formula-fed infants. Moreover, the COGNIS study also provided wide information concerning sociodemographic characteristics of participants (paternal age, IQ, educational level, and place of residence), which were used as potential confounders, giving it an added value to data obtained from this study.

Current analysis has limitations that must be acknowledged. First, the participants’ dropout at 18 months follow-up was 35.91%. Nevertheless, statistical power reached to detect a minimum difference of 0.7 SD was 85%, enough to detect relevant differences in outcomes analyzed in this study between groups. In addition, due to the study design, the BF group was not randomized in contrast to both SF and EF groups, which could explain differences in maternal IQ and parental educational levels [45].

On the other hand, the findings obtained for cVEPs should be taken with caution. This outcome is difficult to assess at these ages, particularly at 3 months of age, and especially the amplitudes and latencies at 7½′ of arc (the perceptual limit of sub-retinal stimulation). As a consequence, a small number of infants showed a response to these minimum stimuli.

## 5. Conclusions

Results obtained in the COGNIS study support that the new experimental infant formula enriched with functional nutrients seemed to have a similar effect during the first 18 months of life on growth patterns compared to the gold standard breastfeeding. Interestingly, brain maturation, assessed as visual function, was improved in infants fed with experimental formula compared to those who received standard formula, mimicking breastfed infants. It is necessary to carry out more randomized clinical trials with long-term monitoring to establish the relationship between postnatal supplementation of the infant and the short- and long-term impact on infant brain development, cognitive performance, and growth.

## Figures and Tables

**Table 1:** Classification of quality of general movements (GM’s)**.**

	*GM’s Classification*		
	Complexity	Variation	Fluidity
**Normal-optimal**	+++	+++	+
**Normal-suboptimal**	++	++	-
**Moderately abnormal**	+	+	-
**Definitively abnormal**	-	-	-

GM’s: general movements; +++: abundantly present; ++: sufficiently present; +: present; -: virtually absent or absent.

**Table 2:** Characteristics of the COGNIS study participants.

		BF (*n* = 50)	SF (*n* = 85)	EF (*n* = 85)	*p* ^1^
**Parents Characteristics**					
Maternal age (years)		32 (30–36.25)	32 (24.75–35.25)	30.50 (26.25–34.75)	0.068
Paternal age (years)		35.07 ± 5.01	32.68 ± 6.89	33.31 ± 7.03	0.134
Maternal pBMI (kg/m^2^)		23.90 (21.80–26.16)	24.18 (21.75–27.61)	23.68 (21.14–27.30)	0.842
GA at delivery (weeks)		39.5 (38–40.25)	40 (38–40)	40 (39–40)	0.925
GWG (kg)		6 (4–9)	7 (3.5–10)	6 (3–9.5)	0.781
Siblings	No	28 (56)	33 (38.80)	42 (49.40)	0.128
	Yes	22 (44)	52 (61.20)	43 (50.60)	
Type of delivery	Vaginal	37 (74)	62 (72.90)	60 (70.60)	0.899
	Caesarean	13 (26)	23 (27)	25 (29.40)	
Smoking during pregnancy	Yes	2 (4.70)	13 (18.80)	10 (13)	0.098
Postpartum Depression	Yes	6 (12)	21 (24.7)	22 (26.2)	0.131
Maternal IQ (points)		111 (99–118) ^a^	102 (92–111) ^b^	100 (86–108) ^b^	<0.001
Maternal educational level	Primary	1 (2) ^a^	19 (22.40) ^b^	19 (22.40) ^b^	<0.001
	Secondary	5 (10) ^a^	28 (32.90) ^b^	29 (34.10) ^b^	
	VT	16 (32)	15 (17.60)	21 (24.70)	
	University	28 (56) ^a^	23 (27.10) ^b^	16 (18.80) ^b^	
Paternal IQ (points)		108 (99–117)	108 (96–117)	102 (92–111)	0.062
Paternal educational level	Primary	6 (12.80) ^a^	28 (35) ^b^	36 (46.20) ^b^	0.003
	Secondary	11 (23.40)	25 (31.30)	16 (20.50)	
	VT	12 (25.50)	13 (16.30)	12 (15.40)	
	University	18 (38.30) ^a^	14 (17.40) ^b^	14 (17.90) ^b^	
Place of residence	Urban	15 (30)	38 (44.70)	28 (32.90)	0.148
	Rural	35 (70)	47 (55.30)	57 (67.10)	
**Newborn characteristics**					
Sex (boy)		21 (42)	49 (57.6)	51 (60)	0.105
Birth weight (g)		3321.20 ± 431.73	3266.25 ± 459.08	3347.76 ± 486.41	0.513
Birth length (cm)		51 (49–51)	50 (49–52)	51 (49–52)	0.431
Birth HC (cm)		35 (33.25–35)	35 (34–35.5)	34.25 (34–35)	0.481
Breastfeeding (days)		420 (270–540)	8 (0–22)	7 (1–28)	<0.001

Data are presented as *n* (%) for categorical data, mean ± SDs for parametrically distributed data, and median (IQR) for non-parametrically distributed data. ^1^ *p*-values for overall differences between COGNIS study groups. *p*-values were obtained from ANOVA for normally distributed variables, Kruskal–Wallis rank-sum test for non-normal continuous variables, and Chi-square test for categorical variables. Values not sharing the same suffix (ab) were significantly different in the Bonferroni post hoc test. BF: breastfed infants; SF: standard infant formula; EF: experimental infant formula; pBMI: pre-gestational body mass index; IQ: intelligence quotient; VT: vocational training; GWG: gestational weight gain; GA: gestational age; HC: head circumference.

**Table 3:** General movements classification at 2, 3, and 4 months of life in infants participating in the COGNIS study.

GM’s	Moderately Abnormal	Normal-Suboptimal	Normal-Optimal	*p* ^1^
**2 months of life**				
BF (*n* = 31)	10 (32.3)	19 (61.3)	2 (6.5)	0.636
SF (*n* = 70)	23 (32.9)	36 (51.4)	11 (15.7)	
EF (*n* = 77)	29 (37.7)	40 (51.9)	8 (10.4)	
**3 months of life**				
BF (*n* = 32)	10 (31.3)	21 (65.6)	1 (3.1)	0.322
SF (*n* = 59)	11 (18.6)	39 (66.1)	9 (15.3)	
EF (*n* = 61)	17 (27.9)	38 (62.3)	6 (9.8)	
**4 months of life**				
BF (*n* = 37)	15 (40.5)	20 (54.1)	2 (5.4)	0.453
SF (*n* = 62)	21 (33.9)	31 (50)	10 (16.1)	
EF (*n* = 69)	20 (29)	37 (53.6)	12 (17.4)	

Data are presented as *n* (%). ^1^*p*-values for overall differences between COGNIS study groups. *p*-values were obtained from the Chi-square test for categorical variables. GM’s: general movements; BF: breastfed infants; SF: standard infant formula; EF: experimental infant formula.

**Table 4:** Latencies and amplitudes of infant’s P100 wave cortical visual evoked potentials (cVEPs) at 3 and 12 months of age in infants participating in the COGNIS study.

		3 Months of Life				12 Months of Life			
		*n*	X ± SD	*P_unadj_*	*P_adj_*	*n*	X ± SD	*P_unadj_*	*P_adj_*
***Latencies (ms)***									
**P100 120′ of arc**	BF	29	117.48 ± 14.46 ^b^	0.059	0.052	36	106.22 ± 6.70	0.759	0.470
	SF	59	125.68 ± 19.10 ^a,b^			45	106.40 ± 8.83		
	EF	67	127.12 ± 19.27 ^a^			58	107.34 ± 8.22		
**P100 60′ of arc**	BF	29	122.03 ± 17.90 ^b^	0.028	0.012	36	108.69 ± 7.13	0.402	0.225
	SF	58	128.74 ± 16.48 ^a,b^			46	112.00 ± 14.51		
	EF	67	133.16 ± 20.55 ^a^			54	110.80 ± 9.60		
**P100 30′ of arc**	BF	29	127.34 ± 20.85 ^b^	0.029	0.015	36	113.75 ± 7.52	0.186	0.090
	SF	55	137.62 ± 20.43 ^a,b^			43	115.21 ± 11.90		
	EF	65	139.80 ± 21.68 ^a^			57	117.75 ± 11.19		
**P100 15′ of arc**	BF	24	136.7 ± 17.65 ^b^	0.021	0.040	29	116.41 ± 8.04 ^a^	0.004	0.004
	SF	38	143.63 ± 30.31 ^a,b^			28	124.00 ± 13.92 ^b^		
	EF	51	153.16 ± 22.66 ^a^			43	124.91 ± 9.95 ^b^		
**P100 7½′ of arc**	BF	6	161.00 ± 19.89	0.142	0.445	15	128.73 ± 6.72 ^a^	0.061	0.031
	SF	4	183.25 ± 37.21			8	136.38 ± 9.41 ^a,b^		
	EF	6	104.50 ± 92.84			22	136.14 ± 11.28 ^b^		
***Amplitudes (µV)***									
**P100 120′ of arc**	BF	29	23.24 ± 11.95	0.103	0.085	36	21.09 ± 10.58 ^a^	0.007	0.003
	SF	59	18.04 ± 8.62			45	15.77 ± 8.21 ^b^		
	EF	67	20.14 ± 11.54			58	15.86 ± 7.20 ^b^		
**P100 60′ of arc**	BF	29	27.73 ± 14.85 ^a^	0.004	0.007	36	19.40 ± 8.55	0.204	0.189
	SF	58	20.09 ± 10.86 ^b^			46	16.40 ± 8.17		
	EF	67	18.97 ± 11.60 ^b^			54	16.57 ± 8.39		
**P100 30′ of arc**	BF	29	23.64 ± 15.78 ^a^	0.086	0.014	36	15.83 ± 8.82	0.505	0.311
	SF	55	16.52 ± 9.08 ^b^			43	13.28 ± 7.28		
	EF	65	17.04 ± 9.59 ^b^			57	14.33 ± 11.52		
**P100 15′ of arc**	BF	24	19.84 ± 12.57 ^a^	0.046	0.005	29	13.72 ± 10.63	0.201	0.288
	SF	38	13.22 ± 8.51 ^b^			28	10.49 ± 6.01		
	EF	52	12.82 ± 6.97 ^b^			43	12.92 ± 6.68		
**P100 7½′ of arc**	BF	6	13.83 ± 5.35	0.181	0.923	15	14.12 ± 10.37	0.789	0.645
	SF	4	14.28 ± 5.76			8	13.00 ± 5.97		
	EF	5	6.02 ± 10.06			22	12.45 ± 4.53		

Data are presented as means ± SD. *P_unad_*_j_ is ANOVA. Values not sharing the same suffix (ab) were significantly different in a Bonferroni post hoc test. *P_adj_* is ANCOVA for the group differences using the univariate general linear model, including confounder factors: maternal educational level and IQ, and paternal educational level. BF: breastfed infants; SF: standard infant formula; EF: experimental infant formula; ms: milliseconds; µV: microvolts; ′: minutes of visual angle.

## Supplementary Materials

The following are available online at https://www.mdpi.com/2072-6643/11/10/2456/s1; Table S1: Longitudinal study of growth up to 18 months of age in COGNIS infants.

## Statement

The results of this article are likely to be included in the Doctoral Thesis of A.N-R. in the context of the Clinical Medicine and Public Health Doctoral Program at the University of Granada.
