# Supplementary material for: Cortical Visual Evoked Potentials and Growth in Infants Fed with Bioactive Compounds-Enriched Infant Formula: Results from COGNIS Randomized Clinical Trial
Source: Nutrients. 2019 Oct 14;11(10):2456. doi: 10.3390/nu11102456 (PMC6835488; doi:10.3390/nu11102456)
Supplement: Supplementary file 1 [file nutrients-11-02456-s001.pdf]

Supplementary table 1. Longitudinal study of growth up to 18 months of age in COGNIS infants.

		2 mo of life	3 mo of life	4 mo of life	6 mo of life	12 mo of life	18 mo of life	*p*[1]	*p*[2]	*p*[3]
**WAZ**	SF (n=46)	-0,37 ± 0,83	-0,34 ± 0,83	-0,18 ± 0,84	0,08 ± 0,81	0,38 ± 0,85	0,35 ± 0,78	0.000	0.176	0.684
	EF (n=53)	-0,41 ± 0,93	-0,28 ± 0,98	-0,09 ± 1,01	0,06 ± 1,06	0,36 ± 1,16	0,35 ± 1,06			
	BF (n=24)	0,07 ± 0,72	-0,02 ± 0,79	0,01 ± 0,88	0,06 ± 0,97	0,42 ± 0,99	0,38 ± 0,99			
**WLZ**	SF (n=46)	0,54 ± 1,02	0,36 ± 0,90	0,24 ± 0,95	0,47 ± 0,93	0,58 ± 0,86	0,63 ± 0,85	0.014	0.350	0.808
	EF (n=53)	0,48 ± 1,04	0,37 ± 1,16	0,48 ± 1,13	0,47 ± 1,09	0,64 ± 1,08	0,61 ± 0,98			
	BF (n=24)	0,85 ± 0,97	0,55 ± 0,88	0,33 ± 0,92	0,45 ± 1,04	0,78 ± 0,86	0,70 ± 0,76			
**LAZ**	SF (n=46)	-0,80 ± 0,96	-0,74 ± 0,82	-0,46 ± 0,92	-0,35 ± 0,98	-0,10 ± 1,05	-0,23 ± 0,99	0.000	0.158	0.914
	EF (n=53)	-0,79 ± 1,10	-0,67 ± 0,99	-0,59 ± 0,98	-0,38 ± 0,99	-0,25 ± 1,10	-0,18 ± 1,04			
	BF (n=24)	-0,55 ± 0,82	-0,50 ± 0,89	-0,30 ± 0,93	-0,35 ± 0,99	-0,33 ± 1,10	-0,27 ± 1,24			
**BAZ**	SF (n=46)	0,10 ± 0,88	0,11 ± 0,88	0,12 ± 0,91	0,37 ± 0,93	0,60 ± 0,88	0,69 ± 0,89	0.000	0.176	0.684
	EF (n=53)	0,04 ± 0,95	0,14 ± 1,12	0,34 ± 1,11	0,37 ± 1,11	0,68 ± 1,07	0,66 ± 0,94			
	BF (n=24)	0,53 ± 0,87	0,33 ± 0,85	0,24 ± 0,92	0,35 ± 1,06	0,84 ± 0,84	0,79 ± 0,68			

Data are presented as means ± SD of Z-Scores. p-values were obtained from Generalized linear mixed model of repeated measures**.** *p*[1] are differences between time points; *p*[2] differences between time points according to the COGNIS groups; *p*[3] are differences longitudinally between COGNIS groups. Mo: Months; WAZ: Weight/Age; WLZ: Weight/Length; LAZ: Length/Age; BAZ: BMI/Age; SF: Standard infant Formula; EF: Experimental infant Formula; BF: Breastfeeding.
